# Biological Control Efficacy and Action Mechanism of *Klebsiella pneumoniae* JCK-2201 Producing Meso-2,3-Butanediol Against Tomato Bacterial Wilt

**DOI:** 10.3389/fmicb.2022.914589

**Published:** 2022-07-14

**Authors:** Bora Kim, Ae Ran Park, Chan Woo Song, Hyohak Song, Jin-Cheol Kim

**Affiliations:** ^1^Department of Agricultural Chemistry, Institute of Environmentally Friendly Agriculture, College of Agriculture and Life Science, Chonnam National University, Gwangju, South Korea; ^2^Research and Department Center, GS Caltex Corporation, Daejeon, South Korea

**Keywords:** *Klebsiella pneumoniae*, tomato bacterial wilt, 2,3-butanediol, induced resistance, qRT-PCR

## Abstract

Bacterial wilt caused by *Ralstonia solanacearum* is a fatal disease that affects the production of tomatoes and many other crops worldwide. As an effective strategy to manage bacterial wilt, biological control agents using plant growth-promoting rhizobacteria (PGPR) are being developed. In this study, we screened 2,3-butanediol (BDO)-producing PGPR to control tomato bacterial wilt and investigated the action mechanism of the disease control agent. Of the 943 strains isolated from soil, *Klebsiella pneumoniae* strain JCK-2201 produced the highest concentration of 2,3-BDO. The culture broth of *K. pneumoniae* JCK-2201 did not show any direct activity on *R. solanacearum in vitro*, but a 100-fold dilution effectively controlled tomato bacterial wilt with a control value of 77% *in vivo*. Fermentation utilizing *K. pneumoniae* JCK-2201 was optimized to produce 48 g/L of meso-2,3-BDO, which is 50% of the sucrose conversion efficiency. In addition, the control efficacy and mechanism of meso-2,3-BDO produced by JCK-2201 in tomato bacterial wilt were determined by comparative analysis with *Bacillus licheniformis* DSM13 producing meso-2,3-BDO and *B. licheniformis* DSM13 *ΔalsS* that did not produce 2,3-BDO, as the step of converting pyruvate to α-acetolactate was omitted. Tomato seedlings treated with the *K. pneumoniae* JCK-2201 (500-fold dilution) and *B. licheniformis* DSM13 (100-fold dilution) culture broth produced meso-2,3-BDO that significantly reduced *R. solanacearum*-induced disease severity with control values of 55% and 63%, respectively. The formulated meso-2,3-BDO 9% soluble concentrate (SL; 1,000-fold dilution) showed 87% control against tomato bacterial wilt in the field condition. *Klebsiella pneumoniae* JCK-2201 and *B. licheniformis* DSM13 treatment induced the expression of plant defense marker genes, such as *LePR1*, *LePR2*, *LePR5*, *LePR3*, and *PI-II*, in the salicylic acid and jasmonic acid signaling pathways at 4 days after inoculation. These results show that 2,3-BDO-producing bacteria and 2,3-BDO are potential biological control agents that act through induction of resistance for controlling tomato bacterial wilt.

## Introduction

*Ralstonia solanacearum* is one of the most important phytopathogenic bacteria that attack many crops and native plants in tropical and subtropical climates worldwide and has a very extensive host range. It enters the roots from the soil and attacks the host’s vascular structures, causing the plant to wilt and perish (Denny, 2007; Sebastià et al., 2021). Tomato bacterial wilt caused by *R. solanacearum* affects the youngest leaves and causes yield loss through wilting and stunted growth (Ji et al., 2005). Various strategies, such as soil improvement, field sanitation, breeding of resistant varieties, and improvement of cropping systems, have been used to control this disease (Xue et al., 2013; Zheng et al., 2019). However, this disease is very difficult to control owing to the high variability of the pathogen, limited chemical control potential, high viability rate of the pathogen in various environments, and broad host range (Mohammed et al., 2020). Therefore, sustainable, economical, and environment-friendly biological controls have been proposed as alternatives over the past few decades (Van Lenteren, 2001).

Among the biological control agents, plant growth-promoting rhizobacteria (PGPR) have been reported as potential candidates as they promote plant growth, improve quality, and directly or indirectly control diseases (Jetiyanon and Kloepper, 2002). PGPR, such as the genera *Pseudomonas*, *Klebsiella*, *Enterobacter*, *Azospirillum*, *Burkholderia*, *Bacillus*, and *Serratia*, promote plant growth and control plant diseases (Glick et al., 1998; Shaharoona et al., 2007; Mazumdar et al., 2018). *Klebsiella pneumoniae* HR1 reduces the incidence of root rot-causing *Macrophomina phaseolina* in *Vigna mungo* L. and significantly increases the germination rate, shoot and root length, and shoot and root dry weight in the treated plants (Dey et al., 2019). *Klebsiella pneumoniae* NG14 has nitrogen-fixing activity and colonizes the root surface and vascular tissue of rice (Liu et al., 2011). *Klebsiella jilinsis* 2N3 induces resistance to corn leaf blight caused by *Setosphaeria turcica*, a hemibiotrophic ascomycete fungal pathogen, and promotes maize growth (Zhang et al., 2021). In addition, *Bacillus cereus* Sneb 560, *B. subtilis* Sneb 815, *Pseudomonas putida* Sneb 821, *P. fluorescens* Sneb 825, and *Serratia proteamaculans* Sneb 821 isolated from the rhizosphere soil show strong larvicidal and nematicidal activity *in vitro* and promote higher root and shoot growth compared to the control when tomato seeds were treated in pot experiments (Zhao et al., 2018). Taken together, PGPR isolates can be used as potential biological control agents against various plant diseases in the future.

All plants have defense mechanisms against pathogens. In general, induced resistance acts systemically because it is not limited to the infected plant parts, and this is commonly referred to as systemic acquired resistance (SAR; Van Loon et al., 1998). SAR involves the accumulation of the signal molecule salicylic acid (SA) and pathogenesis-related proteins (PRs) and confers persistent and broad-spectrum resistance. Induced systemic resistance (ISR) relies on pathways regulated by jasmonic acid (JA) and ethylene (ET). Some PRs hydrolyze fungal cell walls with chitinase and β-1,3-glucanases and are used as markers for SA-associated necrosis infection (Yan et al., 2002; Park et al., 2007a). Activation of ISR by beneficial microorganisms in the plant rhizosphere makes plants healthy and is distinct from SAR, which is a response associated with the aerial part (Pieterse et al., 2004).

Induced resistance occurs when the plant’s defense mechanism induced by PGPR, such as *Pseudomonas*, *Klebsiella*, and *Bacillus*, resists infection and does not cause visible damage to the root system. *Klebsiella pneumoniae* SnebYK treatment promotes soybean growth and induces systemic resistance by expression of *PR1*, *PR2*, *PR5*, and *PDF1.2* to inhibit the invasion and development of *Heterodera glycines* (Liu et al., 2018). Some strains of PGPR also play an important role in plant disease control by enhancing ISR *via* the production of volatile organic compounds (VOCs), such as acetoin and 2,3-butanediol (BDO; Kirankumar et al., 2008; Lee et al., 2012). 2,3-BDO production in microorganisms was first reported in 1906 from *K. pneumoniae* (Harden and Walpole, 1906). Moreover, strains of the genera *Klebsiella* and *Enterobacter* primarily produce (2S,3S)-BDO (known as dextro-2,3-BDO) and (2R,3S)-BDO (known as meso-2,3-BDO), whereas the genus *Bacillus* produces (2R,3R)-BDO (known as levo-2,3-BDO) and meso-2,3-BDO (Ji et al., 2011). α-Acetolactate synthase (ALS), acetolactate decarboxylase (ALDC), and acetoin reductase (AR) are involved in the 2,3-BDO production process. These three enzymes catalyze the production of 2,3-BDO in successive metabolic conversion steps: pyruvate to ALS, ALS to acetoin, and acetoin to 2,3-BDO (Song et al., 2019).

Previous reports have demonstrated that activating SAR and ISR is a promising and effective approach to control a wide range of plant diseases (Kong et al., 2018; Liu et al., 2018). Furthermore, because PGPR strains do not normally exhibit direct activity unlike chemical pesticides, they can be controlled without selective pressure and appear to be environment-friendly (Vallad and Goodman, 2004). *Klebsiella pneumoniae*, a PGPR strain, produces meso-2,3-BDO and induces resistance to the symptoms of wilt and root rot disease and sheath blight of rice (Ji et al., 2014). To the best of our knowledge, previous studies have not investigated the induced resistance effect of *K. pneumoniae* on tomato bacterial wilt and the control ability of meso-2,3-BDO on tomato bacterial wilt in the field.

During the screening of antagonistic PGPR with high induced resistance activity, we found that *K. pneumoniae* JCK-2201 strain produces a high amount of meso-2,3-BDO and can effectively suppress the development of tomato bacterial wilt even though it was not active to *R. solanacearum in vitro* bioassay. In order to elucidate the role of meso-2,3-BDO in the induced resistance activity of *K. pneumoniae* JCK-2201, we tried to make mutants that do not produce meso-2,3-BDO. However, we failed to make the mutants. Therefore, we tried to make meso-2,3-BDO knockout mutants using *Bacillus licheniformis* DSM13 which can produce meso-2,3-BDO (Veith et al., 2004). It also did not show any *in vitro* antibacterial activity against *R. solanacearum* like *K. pneumoniae* JCK-2201. Accordingly, the objectives of this study were to (1) optimize 2,3-BDO production by *K. pneumoniae* JCK-2201, selected through the Voges–Proskauer (VP) test, (2) evaluate the *in vivo* effects of *K. pneumoniae* JCK-2201, *B. licheniformis* DSM13, and *B. licheniformis* DSM13 *ΔalsS* strains on tomato bacterial wilt, (3) investigate the effect of meso-2,3-BDO in the tomato field, and (4) elucidate the action mechanism against tomato bacterial wilt by qRT-PCR analysis of defense-related genes.

## Materials and Methods

### Isolation of Bacterial Strains From Soil

To isolate acetoin-producing bacteria, we collected soil samples from several sampling sites, *viz.*, Gwang-ju campus of Chonnam National University (35.176°N, 126.906°E), Mt. Mudeung (35.134°N, 126.989°E), Mt. Naejang (35.486°N 126.883°E), and Gok-seong area (35.282°N, 127.292°E), in the South Korea. Each soil sample (1 g) was suspended in 9 ml of sterile distilled water and shaken for 10 min. The solution was then serially diluted to 10^−7^ in 10-fold increments. A 100-μl aliquot of the diluted soil was plated on tryptic soy agar (TSA; Becton, Dickinson and Co., Sparks, MA, United States) plates. Plates were incubated at 30°C for 1–3 days. A single colony was then isolated and incubated in tryptic soy broth (TSB; Becton, Dickinson and Co., Sparks, MA, United States) at 30°C with constant shaking at 150 rpm for 1–2 days and then stored at −80°C in 30% glycerol until use.

### Screening Acetoin-Producing Bacteria

Acetoin-producing bacteria were screened using the VP method that detects acetoin, a precursor of 2,3-BDO (Renna et al., 1993). A single colony isolated from soil samples was inoculated into 500 μl of MR-VP medium (7 g/L peptone, 5 g/L dextrose, 5 g/L dipotassium phosphate) in a 1.7-ml e-tube and cultured for 24 h at 37°C. After 24 h of incubation, the culture broth was used for the VP test to distinguish acetoin-producing cells from the background. Culture broth (100 μl) or acetoin (1 mg/ml; Tokyo Chemical Industry Co., Ltd., Tokyo, Japan) was added to each well, and the VP reaction was induced by sequential addition of 60 μl of 5% 1-naphthol dissolved in ethanol and 20 μl of 40% potassium hydroxide dissolved in distilled water. After mixing for 10 min, the color of the culture broth was noted to analyze the presence of acetoin. Cherry red color indicated a positive result that the culture broth contains acetoin produced by a strain and a yellow–brown color indicated a negative result that the culture broth does not contain acetoin. Each sample was replicated three times.

### *In vitro* Test

Acetoin-producing bacteria were grown in TSA at 37°C for 1–2 days and then cultured in 3 ml of TSB in a 15-ml tube at 37°C with constant shaking at 180 rpm for 12 h. The pre-culture was inoculated at 1% in a basal medium containing 50 g/L glucose, 4 g/L K_2_HPO_4_, 1 g/L triammonium citrate, 4 g/L sodium acetate, 10 g/L yeast extract, and 0.8 g/L MgSO_4_·7H_2_O and incubated at 37°C with constant shaking at 180 rpm for 24 h. The culture broth was then filtered through a 0.2-μm membrane filter (Advantec Toyo Co., Tokyo, Japan) to obtain axenic culture filtrate.

*Ralstonia solanacearum* SL341 was cultured in TSB at 30°C with constant shaking at 150 rpm for 2 days, and the concentration of the culture broth was adjusted to 1 × 10^6^ colony-forming units (CFU)/ml. Acetoin-producing bacteria (1 μl) were transferred to each well of a 96-well plate containing *R. solanacearum* SL341 suspension (99 μl). Samples were tested in two-fold dilutions at concentrations ranging from 10% to 0.078%. Streptomycin sulfate (10 mg/ml) dissolved in distilled water was used as the positive control. Plates were incubated at 30°C for 24 h. The minimum inhibitory concentration (MIC) was defined as the lowest concentration of the culture filtrate that completely inhibited bacterial growth and was observed visually. Three replicates were performed.

### Analysis of Acetoin and 2,3-BDO by HPLC

Concentrations of acetoin and 2,3-BDO were determined using an HPLC system (Shimadzu Corporation, Kyoto, Japan) equipped with a DGU-20A degassing unit, LC-20AT pump, SIL-20A autosampler, CTO-20A column oven, RID-20A refractive index detector, and CBM-20A communications bus module. All acetoin-producing bacterial culture broths were centrifuged at 10,000 rpm for 5 min at 4°C and then filtered through a 0.45-μm filter to remove the debris before injecting it into the HPLC system. The samples were eluted isocratically on Aminex HPX-87H column (7.8 × 300 mm; Bio-Rad Laboratories, Hercules, CA, United States) and maintained at 60°C with 5 mM H_2_SO_4_ in water at a flow rate of 0.6 ml/min.

Standard solutions of acetoin (Tokyo Chemical Industry Co., Japan), levo-2,3-BDO, meso-2,3-BDO, and dextro-2,3-BDO (Sigma-Aldrich, St. Louis, MS, United States) were prepared at a concentration of 1.11, 3.33, 10, 30, and 90 mg/ml, respectively, in water. The solutions were filtered through a 0.45-μm Advantec membrane filter. The calibration curve was plotted by linear regression.

### Identification of *Klebsiella pneumoniae* JCK-2201

Genomic DNA of JCK-2201 was extracted using the Bacterial genomic DNA purification Kit (ELPIS-Biotech, Daejeon, South Korea) according to the manufacturer’s recommendations. This strain was identified by sequence analysis of the 16S rRNA gene. The 16S rRNA gene was amplified by PCR using the specific primer pair set 9F (5′-GAGTTTGATCCTGGCTCAG-3′)/1512R (5′-ACGGCTACCTTGTTACGACTT-3′) and sequenced (Genotech Co., Daejeon, South Korea; Le et al., 2020). Gene sequences were compared to the GenBank database using BioEdit version 5.0.9.1 and aligned using ClustalW. A phylogenetic tree was constructed using the neighbor-joining method with 1,000 bootstrap replicates in MEGA version 6.06 (Chen et al., 2016).

### Bacterial Strains and Culture Conditions

The strains and plasmids used in this study are described in Supplementary Table 1. *Escherichia coli* DH5α (Invitrogen, Carlsbad, CA, United States) was used for DNA cloning, whereas *E. coli* HST04 dam^−^/dcm^−^ (Takara, Kyoto, Japan) was used for the propagation of plasmids to prevent the host restriction–modification system in *B. licheniformis* DSM13 before electrotransformation. For genetic analysis, *B. licheniformis* DSM13 was cultivated in Luria–Bertani (LB) medium at 30°C or 50°C depending on the specific requirement. Kanamycin (Km) was used at final concentrations of 50 μg/ml and 20 μg/ml for *E. coli* and *B. licheniformis*, respectively.

### Construction of Plasmids and Gene Manipulation

pBKN was used for homologous recombination-mediated gene manipulation (Song et al., 2021). For scarless deletion of the *alsS* gene, homologous regions at ~700 base pairs upstream [primers: lf-F (*Nhe*I)/lf-R) and downstream (primers: rt-F/rt-R (*Afl*II)] were subjected to PCR to amplify the homologous fragments H1 and H2, respectively (Supplementary Figure 1). To construct a deletion cassette, fusion PCR was performed using flanking primers with H1 and H2 fragments as the template. The fusion product was then ligated into the *Nhe*I/*Afl*II sites of pBKN to construct the recombinant plasmid, pBKN_*alsS*. pBKN_*alsS* was used for conventional homologous recombination-mediated gene deletion. To generate a gene knockout mutant, *B. licheniformis* strain was electrotransformed with the corresponding gene knockout plasmid by the high-osmolarity transformation protocol (Xue et al., 1999). For the sequential 1st/2nd crossover process, the 1st crossover (integration) of the whole plasmid was triggered at a high temperature (50°C) in the cells based on the unstable characteristics of the pE194^ts^ replication origin. PCR was performed to confirm the 1st crossover mutants using genomic-F/screen-R and screen-F/genomic-R primers. Next, several clones were inoculated in LB broth without antibiotics at 50°C. The cells were then sequentially transferred five to eight times in a fresh LB medium to generate a 2nd crossover mutant. Finally, several clones were plated on an LB agar plate without antibiotics. Km-sensitive gene knockout mutants were then screened by replica plating and PCR.

### Medium Optimization for 2,3-BDO Production

Medium optimization for the production of 2,3-BDO was conducted using the basal medium. Optimum carbon and nitrogen sources were selected and then their optimum contents were determined. *Klebsiella pneumoniae* JCK-2201 was incubated in LB medium at 37°C with constant shaking at 180 rpm. The pre-culture was inoculated at 1% into 125-ml Erlenmeyer flasks containing 20 ml of each medium and then incubated at 37°C with constant shaking at 180 rpm. The amounts of acetoin and 2,3-BDO in the culture broth were determined by HPLC analysis after 24 h of incubation. Seven carbon sources, *viz.*, glucose, sucrose, starch, maltose, lactose, fructose, and xylose, were tested as alternative carbon sources at 50 g/L. The optimal concentration of the selected carbon source for the production of 2,3-BDO by *K. pneumoniae* JCK-2201 was tested at 25, 50, 75, 100, 125, and 150 g/L. Similarly, seven nitrogen sources, *viz.*, beef extract, casamino acid, corn steep liquor, NaNO_3_, NH_4_NO_3_, soybean meal, and yeast extract, were tested to replace yeast extract as alternative nitrogen sources at 10 g/L. The optimal concentration of the selected nitrogen source was investigated at 5, 10, 20, and 40 g/L. Finally, after culturing *K. pneumoniae* JCK-2201 in a medium containing the selected carbon and nitrogen sources, HPLC analysis was performed every 12 h to determine the incubation time.

### *In vivo* Pot Experiment

To perform *in vivo* test against tomato bacterial wilt, we modified the conditions previously described (Lee et al., 2015). “Seokwang” tomato seeds (Farm Hannong Co., Ltd., Seoul, South Korea) were used for the wilt test. Tomato seeds were sown in plug trays (5 × 8 cm, Bumnong Co., Ltd., Seoul, South Korea) using horticulture nursery soil (Bumnong Co., Ltd., Seoul, South Korea) and then cultivated in a culture room at 25 ± 5°C for 4 weeks under a light/dark cycle of 16 h/8 h. Tomato plants were transplanted into 7.5 cm (ø) plastic pots. Tomato bacterial wilt was evaluated using 4th–5th-leaf stage tomato plants.

*Klebsiella pneumoniae* JCK-2201 culture broth, containing basal medium, was centrifuged at 10,000 rpm for 10 min to obtain the supernatant was then diluted to 100-, 500-, 1,000-, and 5,000-fold. Optimal *K. pneumoniae* JCK-2201 culture broth was diluted at 500-fold, and *B. licheniformis* DSM13 and *B. licheniformis* DSM13 *ΔalsS* were prepared by diluting at 100-fold. Meso-2,3-BDO was prepared at concentrations of 1 mM and 0.1 mM. Samples (20 ml) were treated by soil drench 7 and 2 days before inoculation. Sungbocycline (17% oxytetracycline; Sungbo Chemicals Co., Ltd., Seoul, South Korea) was used as the positive control and treated by soil drench 1 day before inoculation at 1,000-fold dilution.

*Ralstonia solanacearum* SL341 was cultured in TSA at 30°C for 3 days, and the cells were then harvested with water. *Ralstonia solanacearum* SL341 cell suspension (20 ml; 1 × 10^8^ CFU/ml) was inoculated by soil drench. The inoculated plants were maintained in the dark for 24 h at 30 ± 3°C and then transferred to an incubation room at 30°C, 12 h of daylight per day, and relative humidity of 75% for 10 days. Disease severity was assessed on a scale of 0–5 described previously (Vu et al., 2017): 0 = no wilted leaves, 1 = one or two wilted leaves, 2 = two or three wilted leaves, 3 = three or four wilted leaves, 4 = more than four wilted leaves, and 5 = a dead plant. Each treatment was replicated three times, and each replicate consisted of three tomato plants. The control value was calculated using the Equation 1.


(1) $$\begin{array}{l} Controlvalue\;\left(\%\right)\\ =\left(\frac{\begin{array}{l} Diseaseseverityofcontrol-\\ diseaseseverityoftreatment \end{array}}{Diseaseseverityofcontrol}\right)\times 100 \end{array}$$

### Field Test

Meso-2,3-BDO-rich [90%] (1.2% levo-2,3-BDO, 93.1% meso-2,3-BDO, 5.7% dextro-2,3-BDO) raw material was produced from GS Caltex Corporation. Meso-2,3-BDO 9% and 0.9% liquid soluble concentrate (SL) formulations were prepared by Yuseong Hwayeon Tech Co., Ltd., Geumsan, South Korea.

The control efficacy of meso-2,3-BDO 9% and 0.9% SL against tomato bacterial wilt was evaluated in a field located in Dunnae-myeon, Hoengseong-gun, Gangwon-do, South Korea. “Seokwang” tomato seeds were sown in a seed tray containing topsoil in a greenhouse and transplanted into a field plot when 6–7 leaves were observed after 5 weeks. The field experiment consisted of 1 m × 7.5-m plots with 0.5-m spacing between the plots, and four replicates of each treatment group were formed using a randomized complete block design. A total of 48 plants were used, with 12 tomato plants per replicate. Bacterial wilt disease was caused naturally because the field had been severely damaged by *R. solanacearum* in the previous season. Tomatoes were treated with the samples four times at 7-days intervals after transplantation by soil drench.

A positive control, Dazomet (98% dazomet; Farm Hannong Co., Seoul, South Korea), was applied at 300 kg per 1 ha of soil 4 weeks before transplantation of tomato plants. Tap water was used as untreated control. Equation 2 was used to investigate the disease incidence 40 days after the first treatment, and Equation 3 was used to calculate the control value.


(2) $$\begin{array}{l} Diseaseincidence\;\left(\%\right)\\ =\left(\frac{Numberofdiseasedplants}{Numberoftestedplants}\right)\times 100 \end{array}$$

(3) Controlvalue%=Disease incidence ofcontrol−disease incidence oftreatmentDisease incidence ofcontrol×100

### Quantitative Real-Time PCR

The effects of *K. pneumoniae* JCK-2201, *B. licheniformis* DSM13, and *B. licheniformis* DSM13 *ΔalsS* on the expression of defense-related genes against tomato bacterial wilt were analyzed using 4–5 leaf stage tomato plants. The culture broth of each strain was treated by soil drench 7 and 2 days before inoculation, and water was used for the untreated control group. Two days after the second treatment, 10 ml of *R. solanacearum* (1 × 10^8^ CFU/ml) was inoculated. Tomato leaves were collected 1, 2, and 4 days after inoculation (DAI). Total RNA of tomato leaves was extracted using the IQeasy™ Plus Plant RNA Extraction Mini Kit (iNtRON Biotechnology, Seong-nam, South Korea) according to the manufacturer’s recommendations. Total RNA was synthesized as cDNA using oligo (dT) primer and SuperScript™ IV reverse transcriptase (Invitrogen Inc., Carlsbad, CA, United States) according to the manufacturer’s instructions. Real-time PCR was performed on a real-time PCR detection system (Bio-Rad CFX 96; Bio-Rad Laboratories, Hercules, CA, United States) using iQ™ SYBR Green Supermix (Bio-Rad Laboratories) according to manufacturer specifications.

PCR primers used in this study were synthesized by Genotech (Daejeon, South Korea; Supplementary Table 2). Ubiquitin (*UBI*) was used as the reference gene (Aimé et al., 2013). Pathogenesis-related protein-1 (*LePR1*), *LePR2*, and *LePR5* were used as SA signaling pathway-related genes (Jia et al., 2013). Plant proteinase inhibitor-II (*PI-II*), *LePR3*, and *LeLOX* are involved in the JA signaling pathway, and *ETR4* is involved in the ET signaling pathway. Catalase (*CAT*) gene is recognized as a reactive oxygen species (ROS) scavenger-associated gene (Mhamdi and Van Breusegem, 2018).

### Statistical Analysis

All statistical analyses were performed using the SPSS statistical analysis software (version 21.0 for Windows; SPSS, IBM Corp., Armonk, NY, United States). Data were expressed as mean ± standard deviation (SD) of replicates, and statistical differences were assessed using one-way ANOVA and determined by Tukey’s HSD test (*p* < 0.05).

## Results

### *Klebsiella pneumoniae* JCK-2201 Strain Selection

Among the 943 strains isolated from soil, 23 strains showed a strong positive effect in the VP medium. Acetoin, levo-2,3-BDO, dextro-2,3-BDO, and meso-2,3-BDO were confirmed by HPLC analysis using a standard compound. However, levo-2,3-BDO and dextro-2,3-BDO could not be distinguished because samples showed the same retention time in the HPLC chromatogram. Among the 23 strains, *K. pneumoniae* JCK-2201 produced the most amount of acetoin and meso-2,3-BDO at 8.62 and 9.65 g/L, respectively (Table 1). In the *in vitro* MIC test against *R. solanacearum* SL341 causing tomato bacterial wilt, *K. pneumoniae* JCK-2201 did not show direct antibacterial activity when the culture broth was treated even at a concentration of 10%.

**Table 1:** Minimum inhibitory concentration (MIC) value against *R. solanacearum* and 2,3-BDO production amounts by the selected JCK strains.

JCK strain no.	MIC value (%)	Amount (g/L)^*^		
		Acetoin	Levo and dextro	Meso
2201	>10	10.93 ± 0.10 a	3.76 ± 0.26 a	7.48 ± 0.32 a
2264	>10	0.32 ± 0.03 i	1.53 ± 0.12 cd	0
2324	2.5	0.89 ± 0.03 fgh	1.61 ± 0.08 bcd	0
2417	>10	4.77 ± 0.09 b	2.12 ± 0.08 bcd	0.22 ± 0.12 b
2508	>10	0.87 ± 0.07 fghi	1.70 ± 0.10 bcd	0
2554	5	0.63 ± 0.05 fgi	1.55 ± 0.19 cd	0
5001	5	1.13 ± 0.07 def	1.69 ± 0.06 bcd	0
5007	5	1.14 ± 0.1 def	2.31 ± 0.10 b	0
5013	5	1.12 ± 0.08 def	1.64 ± 0.05 bcd	0
5021	10	0.68 ± 0.04 fghi	1.64 ± 0.10 bcd	0
5049	5	0.51 ± 0.11 ghi	1.53 ± 0.07 cd	0
5055	10	4.39 ± 0.71 b	1.74 ± 1.00 bcd	0
5061	10	1.64 ± 0.02 cd	1.99 ± 0.09 bcd	0
5079	2.5	0.86 ± 0.04 fghi	1.79 ± 0.12 bcd	0
5085	10	0.60 ± 0.10 fghi	1.65 ± 0.04 bcd	0
5086	2.5	1.51 ± 0.23 cde	1.86 ± 0.17 bcd	0
5091	10	0.36 ± 0.08 i	1.38 ± 0.06 d	0
5150	2.5	1.03 ± 0.06 efg	1.71 ± 0.18 bcd	0
5193	10	1.03 ± 0.07 efg	1.97 ± 0.10 bcd	0
5194	5	0.97 ± 0.07 efg	1.40 ± 0.06 d	0
5195	10	0.94 ± 0.12 fg	1.54 ± 0.07 cd	0
5241	1.25	0.49 ± 0.23 ghi	1.52 ± 0.20 cd	0
5243	5	1.72 ± 0.20 c	1.3 ± 0.12 bc	0

Different letters following the values indicate significant differences between the strains as determined by a Tukey’s HSD test (*p* < 0.05).

* The values are means ± SD (*n* = 3).

### *In vivo* Antibacterial Activity of *Klebsiella pneumoniae* JCK-2201 in Basal Medium Against Tomato Bacterial Wilt

**Figure 1:**
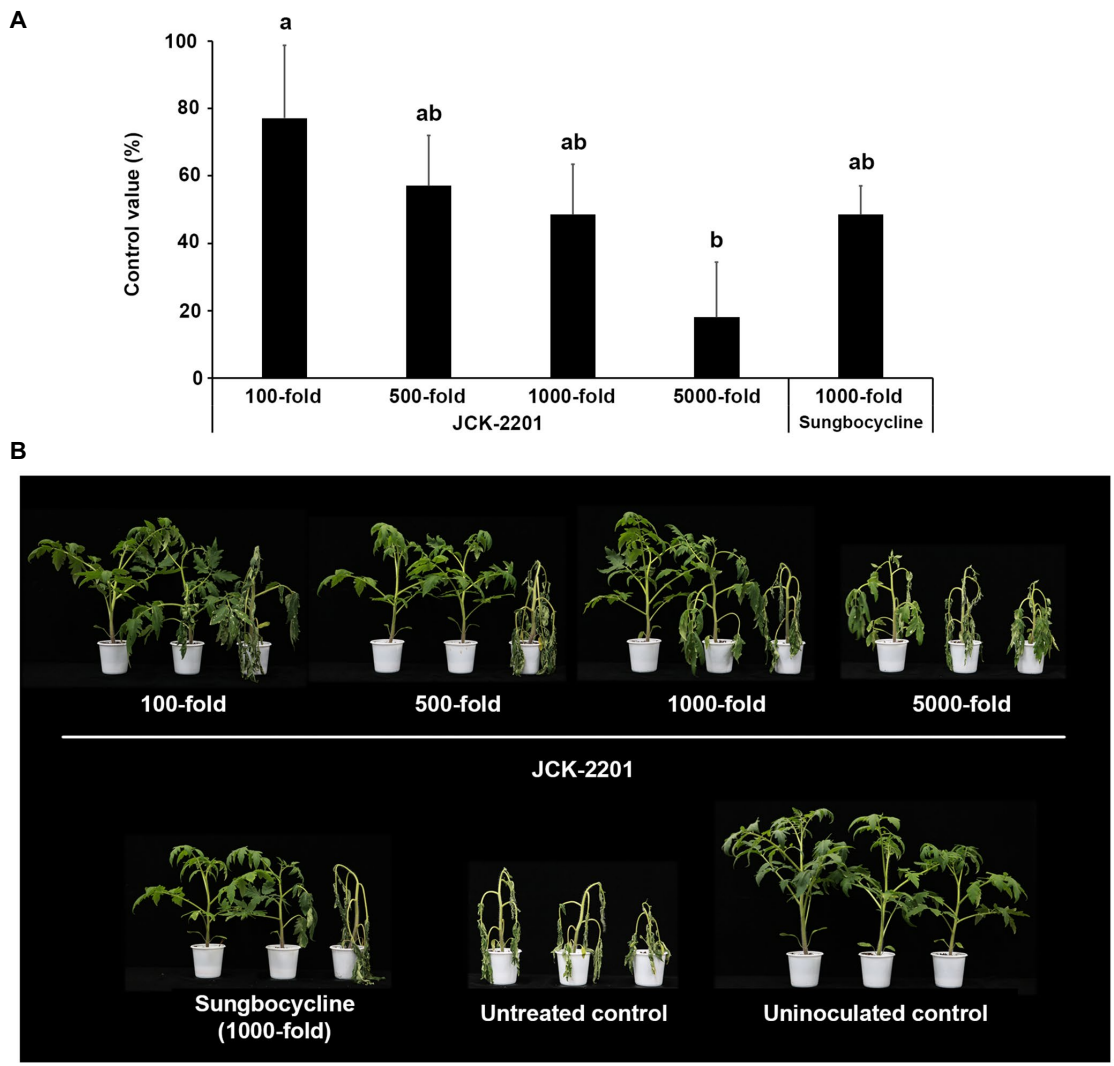
Disease control efficacy of *K. pneumoniae* JCK-2201 culture supernatant on tomato bacterial wilt caused by *R. solanacearum*. **(A)** Control value was investigated 7 days after pathogen inoculation. The data show the means ± SD (*n* = 3). Lower-case letters indicate statistically significant differences as compared to untreated control plants (*p* < 0.05) according to Tukey’s HSD test. Error bars indicate the SDs. **(B)** Representative photographs of tomato seedlings at 7 days after inoculation of *R. solanacearum* by soil drench. As a positive control, plants were treated with Sungbocycline.

To investigate the effect of *K. pneumoniae* JCK-2201 on tomato bacterial wilt, fermentation broth, containing the initial basal medium, of *K. pneumoniae* JCK-2201 was used (Figure 1). In the basal medium, *K. pneumoniae* JCK-2201 produced approximately 9 g/L of meso-2,3-BDO. The culture broth was diluted at 100-, 500-, 1,000-, and 5,000-fold, in which the concentrations of 2,3-BDO were 1, 0.2, 0.1, and 0.02 mM, respectively. Among these four dilution rates, 100-fold dilution showed the highest disease control efficacy against tomato bacterial wilt a control value of 77.14%, which was comparable to that of the positive control Sungbocycline.

### Identification of *Klebsiella pneumoniae* JCK-2201

The phylogenetic tree generated using the 16S rRNA gene sequence of JCK-2201 showed the highest homology with *K. pneumoniae* (Supplementary Figure 2). The JCK-2201 strain was, therefore, identified as *K. pneumoniae* with accession number OM510396 by phylogenetic analysis. Subsequently, JCK-2201 was named *K. pneumoniae* JCK-2201. The strain has been deposited in the Korean Agricultural Culture Collection (KACC, Jeonju, South Korea) as KACC 22683.

### Optimization of the Medium for 2,3-BDO Production by *Klebsiella pneumoniae* JCK-2201

**Figure 2:**
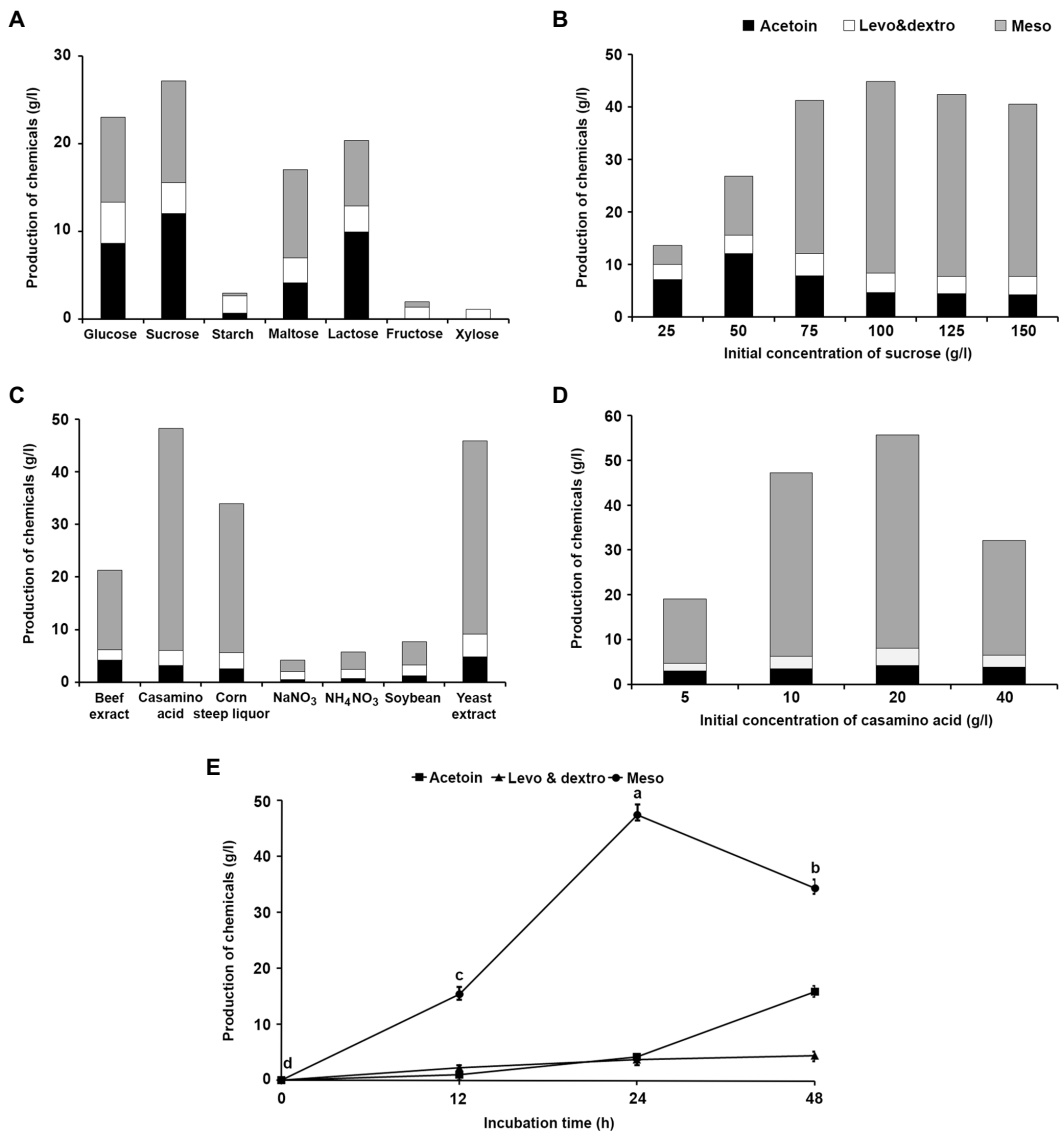
Effect of different carbon and nitrogen sources on the production of acetoin and 2,3-BDO by *K. pneumoniae* JCK-2201. The data show the means ± SD (*n* = 3). Black bar: acetoin, white bar: levo-2,3-BDO and dextro-2,3-BDO, gray bar: meso-2,3-BDO. **(A)** Carbon sources. **(B)** Sucrose concentration. **(C)** Nitrogen sources. **(D)** Casamino acid concentration. **(E)** Comparison of production according to incubation time when cultured in optimum medium containing 100 g/L sucrose and 20 g/L casamino acid. Lower-case letters indicate statistically significant differences as compared to meso-2,3-BDO production by incubation time (*p* < 0.05) according to Tukey’s HSD test.

The medium was optimized for 2,3-BDO production by *K. pneumoniae* JCK-2201. Among the various carbon and nitrogen sources, sucrose and casamino acid were selected as the carbon and nitrogen source, respectively. When 50 g/L of sucrose was used as the carbon source, *K. pneumoniae* JCK-2201 produced acetoin and meso-2,3-BDO at 12.00 and 11.62 g/L, respectively (Figure 2A), and when sucrose was incorporated at 100 g/L, the concentrations of acetoin and meso-2,3-BDO increased to 4.72 and 36.46 g/L, respectively (Figure 2B). When 10 g of casamino acid was used as the nitrogen source, the production of meso-2,3-BDO increased to 42.25 g/L (Figure 2C); when casamino acid was incorporated at 20 g/L, the production of acetoin and meso-2,3-BDO increased to 4.62 and 47.57 g/L, respectively (Figure 2D). In the optimum medium, the production of meso-2,3-BDO reached 47.57 g/L 24 h after inoculation (Figure 2E), after which it decreased. Based on these results, we decided to incubate *K. pneumoniae* JCK-2201 in a medium containing sucrose (100 g) and casamino acid (20 g) at 37°C with constant shaking at 180 rpm for 24 h.

### Disease Control Efficacy of *Klebsiella pneumoniae* JCK-2201, *Bacillus licheniformis* DSM13, and *Bacillus licheniformis* DSM *ΔalsS* Culture Broths Against Tomato Bacterial Wilt in Pot Experiments

**Figure 3:**
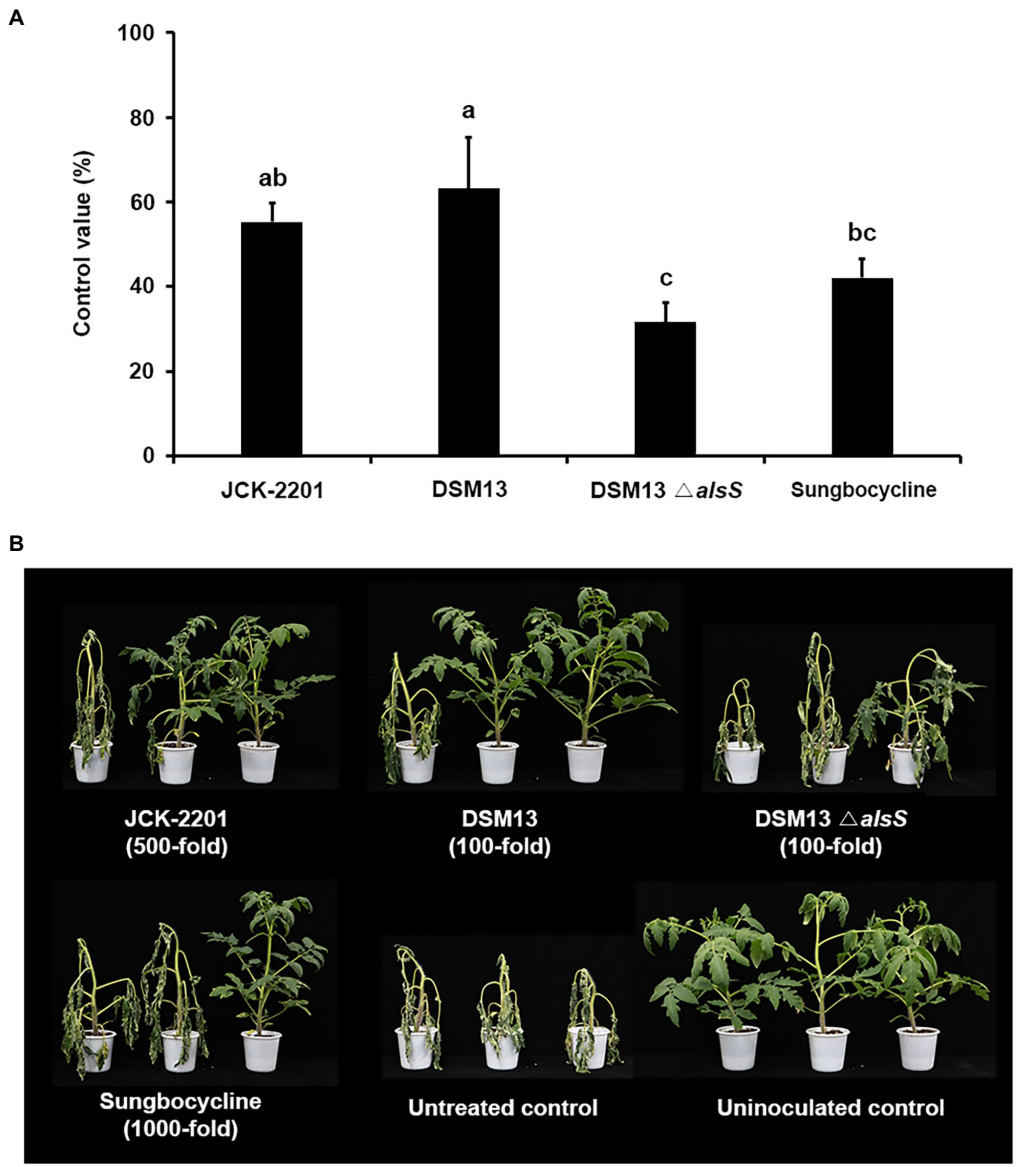
Disease control efficacy of culture supernatant of *K. pneumoniae* JCK-2201, *B. licheniformis* DSM13, and *B. licheniformis* DSM13*ΔalsS* on tomato bacterial wilt caused by *R. solanacearum*. **(A)** Control value was investigated 7 days after pathogen inoculation. The data show the means ± SD (*n* = 3). Lower-case letters indicate statistically significant differences as compared to untreated control plants (*p* < 0.05) according to Tukey’s HSD test. Error bars indicate the SDs. **(B)** Representative photographs of tomato seedlings at 7 days after inoculation of *R. solanacearum* by soil drench. As a positive control, plants were treated with Sungbocycline.

In the optimum medium, *K. pneumoniae* JCK-2201 produced approximately 48 g/L meso-2,3-BDO. *Bacillus licheniformis* DSM13 and *B. licheniformis* DSM13 △*alsS* produced approximately 9 and 0 g/L of meso-2,3-BDO, respectively (Supplementary Figure 3). Treatment with 500-fold diluent of *K. pneumoniae* JCK-2201 culture broth and 100-fold diluent of *B. licheniformis* DSM13 culture broth containing approximately 1 mM meso-2,3-BDO significantly reduced the disease severity by 55.26% and 63.16% compared with the untreated control, respectively (Figure 3). The 100-fold diluent of the culture broth of *B. licheniformis* DSM13 Δ*alsS*, which does not produce meso-2,3-BDO, showed weaker disease control efficacy than the culture broths of *K. pneumoniae* JCK-2201 and *B. licheniformis* DSM13.

**Figure 4:**
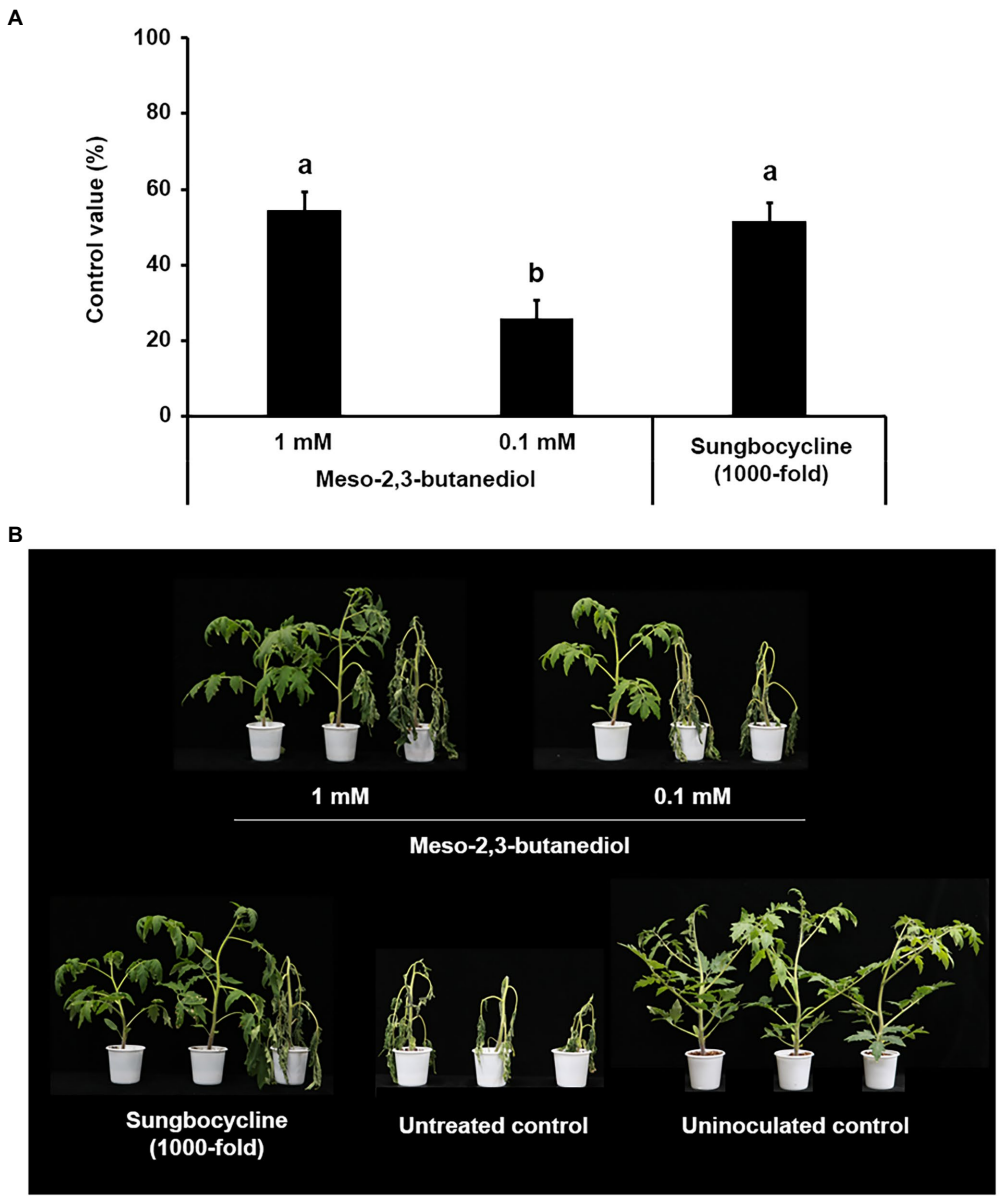
Disease control efficacy of meso-2,3-BDO on tomato bacterial wilt caused by *R. solanacearum*. **(A)** Control value was investigated 7 days after pathogen inoculation. The data show the means ± SD (*n* = 3). Lower-case letters indicate statistically significant differences as compared to untreated control plants (*p* < 0.05) according to Tukey’s HSD test. Error bars indicate the SDs. **(B)** Representative photographs of tomato seedlings at 7 days after inoculation of *R. solanacearum* by soil drench. As a positive control, plants were treated with Sungbocycline.

However, meso-2,3-BDO suppressed the development of tomato bacterial wilt in a dose-dependent manner, with control values of 54.29% and 31.43% at 1 and 0.1 mM, respectively (Figure 4). The control value of 1 mM meso-2,3-BDO was almost similar to that of the 500-fold diluent of *K. pneumoniae* JCK-2201 culture broth and the 100-fold diluent of *B. licheniformis* DSM13 culture broth.

### Disease Control Efficacy of Meso-2,3-BDO SL Formulations Against Tomato Bacterial Wilt in the Field

**Figure 5:**
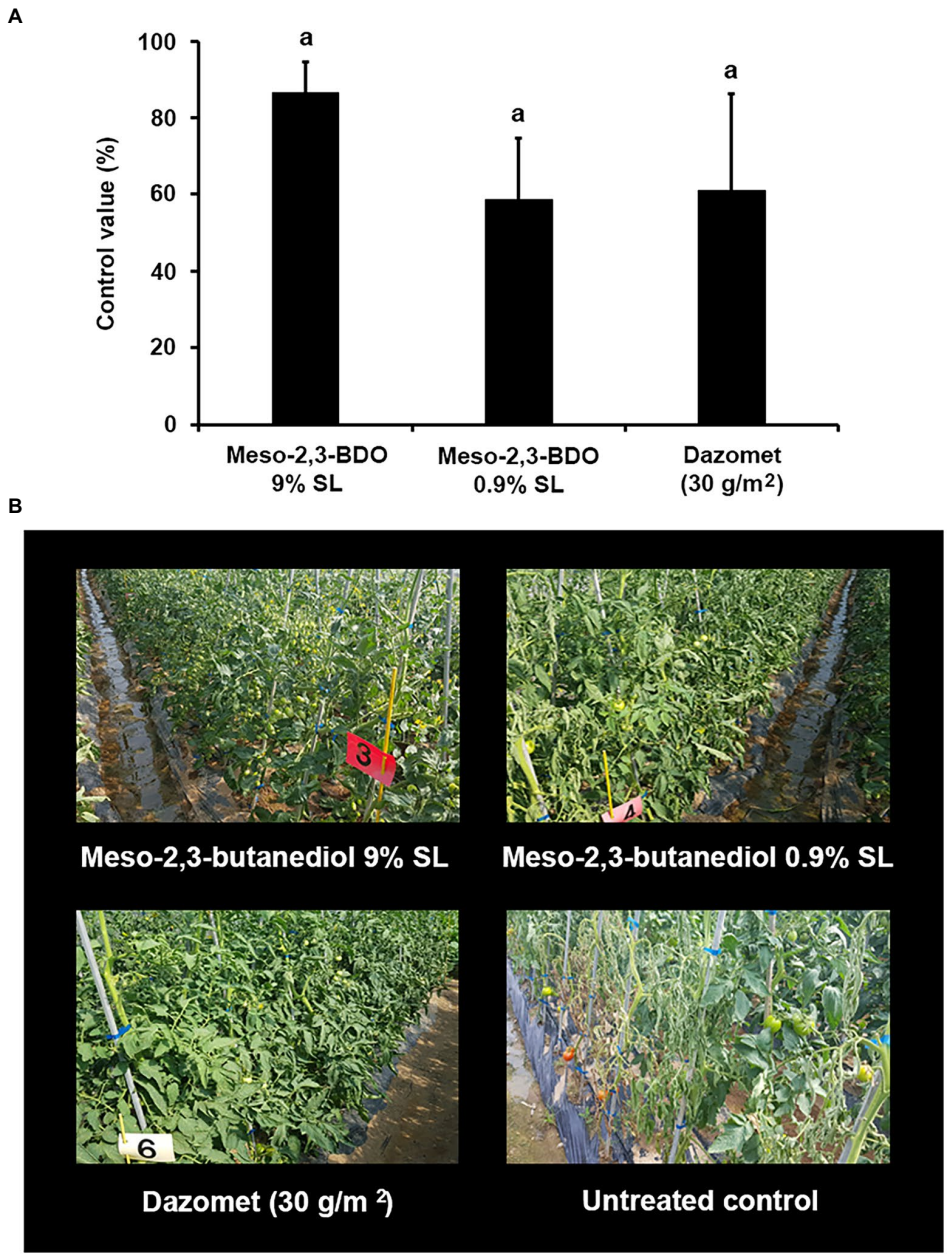
Disease control efficacy of meso-2,3-BDO on tomato bacterial wilt caused by *R. solanacearum* under the field conditions. 2,3-BDO treatment was applied by preparing meso-2,3-BDO in 9% soluble concentrate (SL) and 0.9% SL. After transplantation, the tomatoes were irrigated four times at 7-day intervals at 200 ml of the 1,000-fold diluent of meso-2,3-BDO 9% SL or meso-2,3-BDO 0.9% SL per plant by soil drench. As a positive control, plants were treated with Dazomet at 300 kg per 1 ha. **(A)** Control value was investigated 40 days after pathogen inoculation. The data show the means ± SD (*n* = 4). Lower-case letters indicate statistically significant differences as compared to untreated control plants (*p* < 0.05) according to Tukey’s HSD test. Error bars indicate the SDs. **(B)** Representative photographs of tomato plants after naturally occurring tomato bacterial wilt.

The disease control efficacy of two SL formulations of meso-2,3-BDO (9% SL and 0.9% SL) was evaluated under field conditions. When samples were applied at 1,000-fold dilution by soil drench, the control values were 87% for meso-2,3-BDO (9% SL) and 59% for meso-2,3-BDO (0.9% SL; Figure 5). These results were similar to those of Dazomet, the positive control.

### Expression of Defense-Related Genes Using qRT-PCR

**Figure 6:**
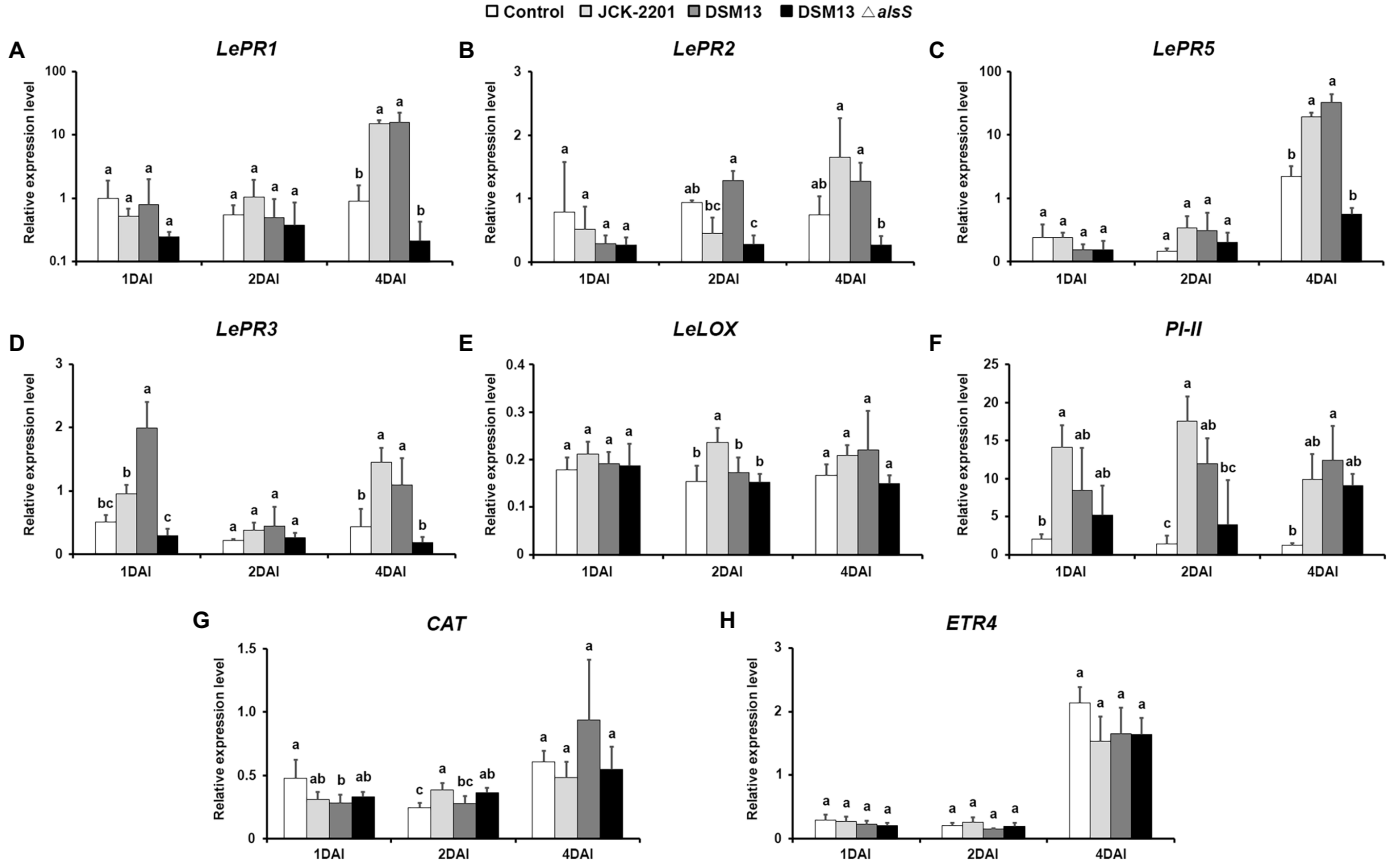
Induction of tomato defense-related gene expression by culture supernatant of *K. pneumoniae* JCK-2201, *B. licheniformis* DSM13, and *B. licheniformis* DSM13*ΔalsS* at 1, 2, 4 days after inoculation. Expression of defense genes of the SA signaling pathway-related genes **(A)** *LePR1*, **(B)** *LePR2*, and **(C)** *LePR5*, JA signaling pathway-related genes **(D)** *LePR3*, **(E)** *LeLOX*, **(F)** *PI-II*, ROS-related gene **(G)** *CAT*, and ET signaling pathway-related gene **(H)** *ETR4* in tomato plants were quantified. The housekeeping gene *LeUBI* was used for normalization. The data show the means ± SD (*n* = 3). Lower-case letters indicate statistically significant differences as compared to untreated control plants (*p* < 0.05) according to Tukey’s HSD test. Error bars indicate the SDs.

To investigate the effect of *K. pneumoniae* JCK-2201 and *B. licheniformis* DSM13 producing 2,3-BDO on the induction of plant defense-related genes, tomato leaves were assessed by qRT-PCR. The expression levels of SA-related pathway genes *LePR1*, *LePR2*, and *LePR5* in the *K. pneumoniae* JCK-2201 (500-fold dilution)-treated leaves were significantly upregulated by 15.14-, 1.65-, and 19.08-fold, respectively, compared to the control group at 4 DAI (Figures 6A–C). The expression levels of *LePR1*, *LePR2*, and *LePR5* in the *B. licheniformis* DSM13 (100-fold dilution) treatment group also increased significantly by 15.96-, 1.27-, and 32.58-fold, respectively (Figures 6A–C). Among the genes involved in the JA-related pathway, *LePR3* and *PI-II*, except for *LeLOX*, were upregulated significantly in the tomato plants at 1 DAI and gradually increased until 4 DAI (Figures 6D–F). In the *K. pneumoniae* JCK-2201-treated group, *LePR3* and *PI-II* expression levels were 1.45- and 9.85-fold higher than those in the untreated group and 1.09- and 12.45-fold higher in the *B. licheniformis* DSM13 group at 4 DAI (Figures 6D,F). Conversely, *CAT* and *ETR4* were not significantly expressed after inoculation (Figures 6G,H).

## Discussion

Acetoin is a flavor compound present in oils, dairy products, and some fruits. Moreover, it is a physiological metabolite produced by many microorganisms and a precursor to 2,3-BDO (Xu et al., 2011). In this study, we selected acetoin-producing bacteria through the VP test and confirmed the production of acetoin and 2,3-BDO by HPLC analysis. The selected strain *K. pneumoniae* JCK-2201 produced acetoin and meso-2,3-BDO but showed no *in vitro* antibacterial activity against *R. solanacearum*. Some PGPR strains control plant diseases by the induced resistance mechanism without directly affecting the pathogens (Vallad and Goodman, 2004). Park et al. (2007b) reported that *Bacillus vallismortis* EXTN-1 does not exhibit direct *in vitro* antibiosis against *R. solanacearum* but effectively inhibits tomato bacterial wilt by inducing systemic resistance *in vivo* and significantly hampering the movement of *R. solanacearum*. Therefore, we predicted that *K. pneumoniae* JCK-2201 potentially induced resistance against plant diseases. To this end, we performed an *in vivo* experiment on tomato bacterial wilt and demonstrated that a 100-fold dilution of *K. pneumoniae* JCK-2201 effectively inhibited tomato bacterial wilt to a control value of 77%.

2,3-BDO is produced by several microorganisms, including *K. pneumoniae*, *B. licheniformis*, and *Paenibacillus polymyxa* (Nilegaonkar et al., 1996; Shrivastav et al., 2013). Generally, *K. pneumoniae* produces meso-2,3-BDO with a small amount of dextro-2,3-BDO, and *B. licheniformis* usually produces a mixture of meso-2,3-BDO and levo-2,3-BDO (Chen et al., 2014; Qiu et al., 2016). 2,3-BDO is used as a promising chemical in a wide range of applications owing to its low toxicity and higher yield in microbial systems (Conveerti and Perego, 2002). *Klebsiella* has the advantage of easy and rapid proliferation in a simple medium. It has also been reported that 1,3-propanediol production is reduced under microaerobic and low pH conditions and sugar is primarily directed for the production of 2,3-BDO (Biebl et al., 1998). Therefore, we conducted an experiment to select the optimal medium to increase the production of 2,3-BDO. When *K. pneumoniae* JCK-2201 was cultured in flasks for 24 h using 100 g/L sucrose and 20 g/L casamino acid, meso-2,3-BDO was produced at the maximum concentration of approximately 48 g/L. *Klebsiella pneumoniae* SDM produces a maximum of 36.7 g/L 2,3-BDO in flasks containing optimal medium when incubated at 37°C with constant shaking at 200 rpm for 38 h (Ma et al., 2009). After 10 h of batch fermentation using glucose as the carbon source, the maximum production of 2,3-BDO by *K. pneumoniae* DSM 2026 and *K. oxytoca* ATCC 43863 were 17.6 and 10.9 g/L, respectively (Cho et al., 2012). *Klebsiella* sp. XMR21 produces the highest amount of 2,3-BDO (14.5 g/L) after 48 h when sucrose was used as a substrate among the four carbon sources, *viz.*, glycerol, xylose, glucose, and sucrose (60 g/L; Xin et al., 2015). Thus, *K. pneumoniae* JCK-2201 produced higher amounts of 2,3-BDO compared to that reported in previous studies. Sucrose as the carbon source can provide a sustainable supply at a lower production cost and is more environment-friendly than glucose (Ji et al., 2011; Zeng and Sabra, 2011).

However, it could not be confirmed whether 2,3-BDO is responsible for the disease control efficacy of *K. pneumoniae* JCK-2201. Therefore, we prepared a mutant strain in which *alsS* was deleted using the *B. licheniformis* DSM13 strain, which produces meso-2,3-BDO, to investigate the role of 2,3-BDO. The fermentation broth diluents of *K. pneumoniae* JCK-2201 and *B. licheniformis* DSM13 showed comparable and higher disease control efficacy against tomato bacterial wilt compared to the positive control of Sungbocycline. The two samples also had significantly higher disease control efficacy than the *B. licheniformis* DSM13 *ΔalsS* strain. Kang et al. (2021) reported that deletion of *alsS* from 2,3-BDO-producing *Saccharomyces cerevisiae* CBMMP1P2 blocked the 2,3-BDO production pathway. Indeed, the *B. licheniformis* DSM13 *ΔalsS* strain, which is the knocked out α-acetolactate synthase *B. licheniformis* DSM13 strain, produced neither acetoin nor 2,3-BDO and showed very low disease control efficacy against tomato bacterial wilt. Volatile metabolites, such as acetoin and 2,3-BDO, induce plant defense mechanisms that help plants overcome bacterial diseases and promote plant growth (Ryu et al., 2004; Magno-Pérez-Bryan et al., 2015). These reports confirm that the disease control efficacy of *K. pneumoniae* JCK-2201 and *B. licheniformis* DSM13 was due to induced resistance by 2,3-BDO. Because *K. pneumoniae* JCK-2201 and *B. licheniformis* DSM13 produced small amounts of levo-2,3-BDO and dextro-2,3-BDO rather than meso-2,3-BDO, we expect that the effects of *K. pneumoniae* JCK-2201 and *B. licheniformis* DSM13 on tomato bacterial wilt is primarily related to meso-2,3-BDO. Meso-2,3-BDO production at 500-fold dilution of *K. pneumoniae* JCK-2201 and 100-fold dilution of *B. licheniformis* DSM13 was approximately 1 mM. In this study, when meso-2,3-BDO was applied at 1 and 0.1 mM, its control values against tomato bacterial wilt were 54 and 31%, respectively. Levo-2,3-BDO treatment reduces diseased leaf area of *Agrostis stolonifera* by 20%–40% against *Microdochium nivale*, *Rhizoctonia solani*, or *Sclerotinia homoeocarpa* (Cortes-Barco et al., 2010). Meso-2,3-BDO treatment also significantly reduced the incidence of naturally occurring cucumber mosaic virus and tobacco mosaic virus compared to untreated controls in a field experiment (Kong et al., 2018). Although the effect of 2,3-BDO on various plant diseases has been reported, its effect on tomato bacterial wilt has not been investigated yet under field conditions. Therefore, we evaluated the disease control efficacy of meso-2,3-BDO against tomato bacterial wilt under field conditions. Meso-2,3-BDO (1 mM) effectively inhibited the development of tomato bacterial wilt at a control value of 87%, with a higher efficacy than dazomet (used as the positive control). This effect of 2,3-BDO treatment is not direct and may be due to the induced resistance effect by the expression of plant defense marker genes in the SA, JA, and ET signaling pathways (Kong et al., 2018). Therefore, we investigated the action mechanism of these genes.

It has been reported that the VOCs from the PGPR strain *P. polymyxa* E681 induce ISR *via* SA and/or JA signaling pathways against *P. syringae* by priming the defense genes *PR1*, *ChiB*, and *VSP2* (Lee et al., 2012). In our experiment, *LePR1*, *LePR2*, and *LePR5* were induced by both *K. pneumoniae* JCK-2201 and *B. licheniformis* DSM13 treatments than by *B. licheniformis* DSM13 *ΔalsS* and untreated control at 4 DAI. The results suggest that meso-2,3-BDO produced by *K. pneumoniae* JCK-2201 and *B. licheniformis* DSM13 activated the SA signaling pathway in tomato after pathogen infection. Similarly, 1 mM 2,3-BDO treatment induced resistance against *R. solanacearum* at 3 DAI, as evidenced by the expression of SA signaling-related PR genes, such as *CaPR2*, *CaSAR8*.2, and *CaPAL* (Yi et al., 2016). Moreover, our results showed that *K. pneumoniae* JCK-2201 and *B. licheniformis* DSM13 treatment induced the expression of JA pathway-related genes, such as *LePR3* and *PI-II*, than that by *B. licheniformis* DSM13 *ΔalsS* and untreated control at 1 DAI. However, *LeLOX* expression was not statistically significant. These results were consistent with the report by Park et al. (2018) that 2,3-BDO produced by *P. polymyxa* DSM365 increases the expression levels of PR protein-encoding genes, such as *PR1*, *PR3*, *PR4-b*, *PR5*, and *PR6*, in tobacco at 48 h after *Phytophthora parasitica* var. *nicotianae* inoculation. Meso-2,3-BDO 9% SL treatment upregulated the expression of the SA-, JA-, and ET-related pathway genes *AsNPR1*, *AsPR4*, *AsLOX*, *AsAOS1*, and *AsERF* as well as ROS-related genes *AsDHAR*, *AsCAT*, *AsMDHAR*, *AsSOD*, *AsGr*, and *AsPOD*, resulting in reduced incidence of dollar spot disease in creeping bentgrass (Duraisamy et al., 2022). However, the ROS-related gene *CAT* and ET pathway-related gene *ETR4* showed relatively low expression levels or were not statistically significantly upregulated by meso-2,3-BDO in this study. These differences may be due to the different plants used. Consequently, we demonstrated that meso-2,3-BDO produced by *K. pneumoniae* JCK-2201 and *B. licheniformis* DSM13 increased the gene expression of SA- and JA-related pathways after *R. solanacearum* pathogen inoculation, resulting in the induction of SAR and ISR and activating defense-related systems against pathogens.

These findings indicate that the upregulated expression of PR genes and plant defense-related genes are responsible for the reduced incidence of *R. solanacearum*. Plant pathogens are divided into biotrophs and necrotrophs according to their life cycle, and hemi-biotrophs act as biotrophs and necrotrophs according to conditions or life cycle stages (Glazebrook, 2005). In general, plant defense responses include SA-dependent defenses acting on biotrophs and JA- and ET-dependent responses acting on necrotrophs; resistance is induced depending on the attacking pathogen (McDowell and Dangl, 2000). However, one recent study confirmed the resistance to *Plasmodiophora brassicae* in *Arabidopsis* by *ARGAH2* and *THI2.1* gene expression, suggesting that JA-mediated defense contributes to resistance to some biotrophic or antibiotic pathogens (Thaler et al., 2004; Lemarie et al., 2015; Ding et al., 2021). *Klebsiella pneumoniae* JCK-2201 and *B. licheniformis* DSM13 induced resistance to *R. solanacearum* by the involvement of both SA and JA signaling pathways and was demonstrated to be related to meso-2,3-BDO produced by *K. pneumoniae* JCK-2201 and *B. licheniformis* DSM13. *Klebsiella pneumoniae* JCK-2201 and meso-2,3-BDO are potential biopesticides for the control of tomato bacterial wilt, and further studies are required on the development of optimum formulation, toxicity test, and evaluation of their disease control efficacy under various field conditions.

## Conclusion

In the present study, the PGPR strain *K. pneumoniae* JCK-2201 effectively suppressed the development of tomato bacterial wilt, and its disease control efficacy was demonstrated to be primarily because of meso-2,3-BDO production in the fermentation broth. This is the first study on the disease control efficacy of meso-2,3-BDO on tomato bacterial wilt under field conditions. The fermentation broth of *K. pneumoniae* JCK-2201 enhanced the expression of plant defense marker genes in both SA and JA signaling pathways. These results show that 2,3-BDO-producing bacteria and 2,3-BDO are potential biopesticides for controlling tomato bacterial wilt.

## Data Availability Statement

The data presented in the study are deposited in the GeneBank repository, accession number OM510396.

## Author Contributions

BK and J-CK designed this study. BK isolated and identified strain *K. pneumoniae* JCK-2201, determined the optimal medium conditions for 2,3-BDO production, and performed *in vitro* and *in vivo* antibacterial bioassays and field experiments. CS and HS provided 2,3-BDO and produced the *B. licheniformis* DSM13 mutant. BK and AP performed qRT-PCR analyses. BK, AP, and J-CK wrote and revised the manuscript. All authors contributed to the article and approved the submitted version.

## Conflict of Interest

CS and HS were employed by the GS Caltex Corporation of South Korea.

The remaining authors declare that the research was conducted in the absence of any commercial or financial relationships that could be construed as a potential conflict of interest.

## Publisher’s Note

All claims expressed in this article are solely those of the authors and do not necessarily represent those of their affiliated organizations, or those of the publisher, the editors and the reviewers. Any product that may be evaluated in this article, or claim that may be made by its manufacturer, is not guaranteed or endorsed by the publisher.

## Supplementary Material

The Supplementary Material for this article can be found online at: https://www.frontiersin.org/articles/10.3389/fmicb.2022.914589/full#supplementary-material
