# Supplementary material for: Biological Control Efficacy and Action Mechanism of Klebsiella pneumoniae JCK-2201 Producing Meso-2,3-Butanediol Against Tomato Bacterial Wilt
Source: Front Microbiol. 2022 Jul 14;13:914589. doi: 10.3389/fmicb.2022.914589 (PMC9333516; doi:10.3389/fmicb.2022.914589)
Supplement: Supplementary file 1 [file Data_Sheet_1.docx]

Supplementary Material

# Supplementary Figures and Tables

## Supplementary Figures


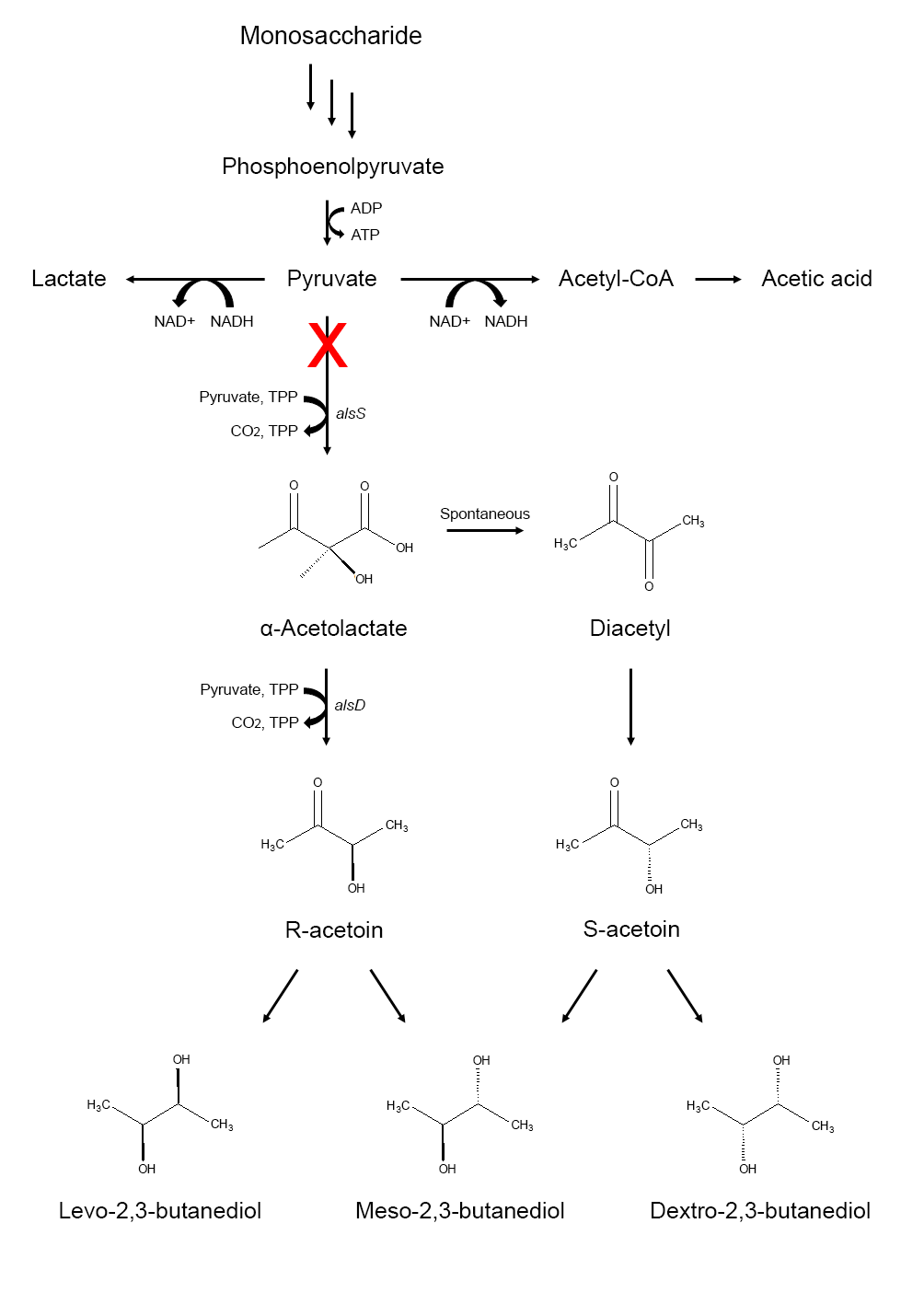


**Supplementary Figure 1.** Metabolic pathway for synthesis of acetoin and 2,3-butanediol optical. Red cross marks indicate the pathway that deletes the corresponding genes in *B. licheniformis* DSM13.


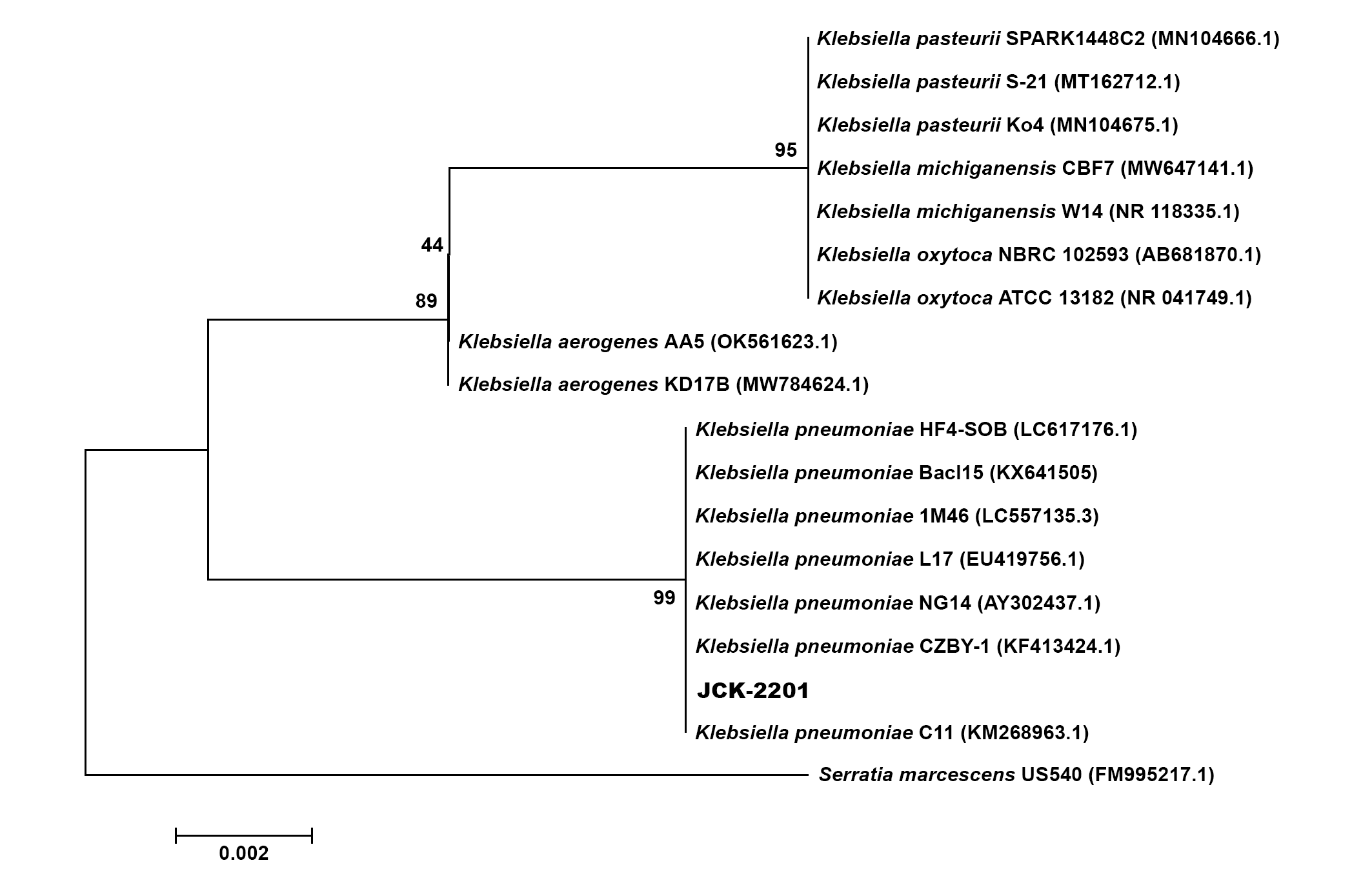


**Supplementary Figure 2.** Neighbor-joining phylogenetic tree of 16S rRNA gene sequences showing relationships between strain JCK-2201. *Serratia marcescens* US540 is used as an outgroup. The sequences were aligned using the CLUSTAL W and were constructed as an integrated neighbor-joining and the Kimura 2-parameter model in the MEGA 6.06 program. The number of nodes is the percentage of bootstrap values obtained by repeating the analysis 1,000 times. The GeneBank accession numbers of each strain are indicated in parentheses. The scale bar represents 1% nucleotides sequence divergence of the 16S rRNA sequence.


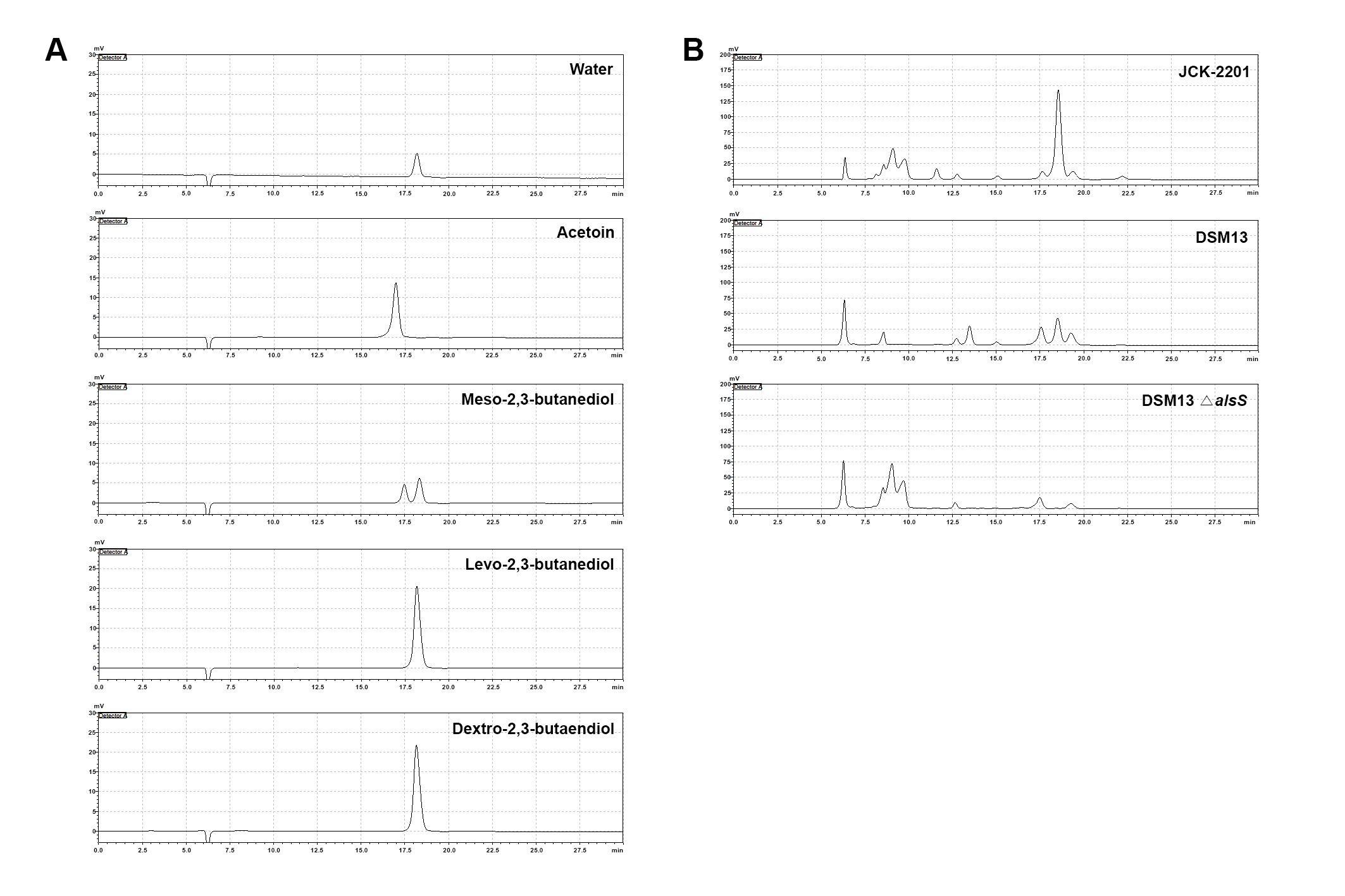


**Supplementary Figure 3.** HPLC chromatogram of acetoin and 2,3-butanediol. (A) Acetoin and three streoiosomers: meso-2,3-butanediol, levo-2,3-butaendiol, and dextro-2,3-butaendiol. (B) Comparison of production amount of acetoin and 2,3-butanediol produced by *Klebsiella pneumoniae* JCK-2201, *Bacillus licheniformis* DSM13, and *B. licheniformis* DSM13 *△alsS*.

## Supplementary Tables

**Supplementary Table 1.** Strains and plasmids used in this study

| Strain / plasmid | Description | Source or reference |  |
| --- | --- | --- | --- |
| Strains | |  |  |
| *E. coli* DH5α | | *fhuA2 lac(Δ)U169 phoA glnV44 Φ80' lacZ(Δ)M15 gyrA96 recA1 relA1 endA1 thi-1 hsdR17* | Invitrogen |
| *E. coli* HST04 dam-/dcm- | | *F-, ara, Δ(lac-proAB)[Φ80dlacZΔM15], rpsL (str), thi, Δ(mrr-hsdRMSmcrBC), ΔmcrA, dam, dcm* | Takara |
| *B. licheniformis* DSM13 | | Natural isolate from soil sample | DSMZ^1)^ |
| *B. licheniformis* DSM13 *ΔalsS* | | *alsS* gene knock-out mutant of DSM13 | This study |
| Plasmids | |  |  |
| pBKN | | Km^R^, derivative of pJOE8999, replicable in *E. coli* (pUC ori) and *Bacillus* (temperature-sensitive pE194^ts^ ori)*,* 4.4-kb | (Song et al., 2021) |
| pBKN_alsS | | Km^R^, pBKN containing left (754-bp) and right (747-bp) homologous franking regions of *alsS* gene from *B. licheniformis*, 4.8-kb | This study |
| ^1)^ DSMZ-German Collection of Microorganisms and Cell Culture GmbH  Abbreviation: Km, kanamycin; R, resistance. | | | |

**Supplementary Table 2.** The primers used in this study

| Primers | Primer sequence (5’-3’) |
| --- | --- |
| lf-F (NheI) | TTCTTGGCTAGCCGCTTCTTTGGCAATGACGATCAGCGATTC |
| lf-R | GTTGTGACTGGACATGCTGTGAAGCCCTCACTCCTCCATTTTC |
| rt-F | GAAAATGGAGGAGTGAGGGCTTCACAGCATGTCCAGTCACAAC |
| rt-R (AflII) | CTTATTCTTAAGAAGAAATCTTGCGTATTCGGCAATCTGAGG |
| screen-F | ATGGCGCAGCTCCATTAATCACTC |
| screen-R | GAGACCTGATAGACTTGATCGAGG |
| genomic-F | TATTCTGTAGCTTCCTGGGCGATG |
| genomic-R | GGAACATAGACGATCCTGACTGAC |
| *LePR1* | For GCC AAG CTA TAA CTA CGC TAC CAA C  Rev GCA AGA AAT GAA CCA CCA TCC |
| *LePR2* | For GGA CAC CCT TCC GCT ACT CTT  Rev TGT TCC TGC CCC TCC TTT C |
| *LePR5* | For AGG TGA CAC TAT AGA ATA GGA GAT GCC TGC AAG TAC TGA  Rev GTA CGA CTC ACT ATA GGG ATT CCG GTC TTT AAG TTT GTT ACG |
| *CAT* | For CCC AGT TAA TGC TCC CAA GT  Rev AGG ACG ACA AGG ATC AAA CC |
| *ETR4* | For CTG CAG ATT GGA ATG AAT GG  Rev ATA AGG CAC CGT CAA CAT CA |
| *LOX* | For ATC TCC CAA GTG AAA CAC CAC A  Rev TCA TAA ACC CTG TCC CAT TCT TC |
| *PI-II* | For TGA TGA ACC CAA GGC AAA TA  Rev ACA CAA CTT GAT GCC CAC AT |
| *LePR3* | For AAC TAT GGG CCA TGT GGA AGA  Rev GGC TTT GGG GAT TGA GGA G |
| *LeUBI* | For GGA CGG ACG TAC TCT AGC TGA T  Rev AGC TTT CGA CCT CAA GGG TA |
